# 1-(4,5-Dinitro-10-aza­tricyclo­[6.3.1.0^2,7^]dodeca-2,4,6-trien-10-yl)-2,2,2-trifluoro­ethanone

**DOI:** 10.1107/S1600536808028158

**Published:** 2008-11-22

**Authors:** Hao Xu, Ji-Cai Quan, Jian Xu, Jing Chen, Jin-Tang Wang

**Affiliations:** a College of Science, Nanjing University of Technolgy, Xinmofan Road No.5, Nanjing 210009, People’s Republic of China

## Abstract

In the title compound, C_13_H_10_F_3_N_3_O_5_, a derivative of andrographolide, the five-membered ring adopts an envelope conformation, while the non-planar six-membered ring has a chair conformation. An intra­molecular C—H⋯F hydrogen bond results in the formation of a non-planar six-membered ring adopting a twisted conformation. In the crystal structure, inter­molecular C—H⋯O hydrogen bonds link the mol­ecules into centrosymmetric dimers.

## Related literature

For bond-length data, see: Allen *et al.* (1987 ▶). For ring puckering parameters, see: Cremer & Pople (1975 ▶).
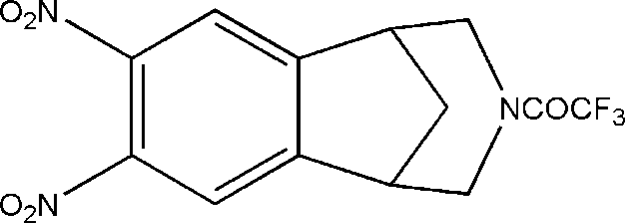


## Experimental

### 

#### Crystal data

C_13_H_10_F_3_N_3_O_5_
*M* *_r_* = 345.24
Monoclinic, 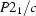

*a* = 9.6400 (19) Å
*b* = 7.7430 (15) Å
*c* = 18.687 (4) Å
β = 96.98 (3)°
*V* = 1384.5 (5) Å^3^
*Z* = 4
Mo *K*α radiation
μ = 0.15 mm^−1^
*T* = 294 (2) K
0.30 × 0.20 × 0.10 mm

#### Data collection

Enraf–Nonius CAD-4 diffractometer
Absorption correction: ψ scan (North *et al.*, 1968 ▶) *T*_min_ = 0.955, *T*_max_ = 0.985
2673 measured reflections
2513 independent reflections
1592 reflections with *I* > 2σ(*I*)
*R*_int_ = 0.052
3 standard reflections frequency: 120 min intensity decay: none

#### Refinement

*R*[*F* ^2^ > 2σ(*F* ^2^)] = 0.061
*wR*(*F* ^2^) = 0.183
*S* = 1.02
2513 reflections
217 parameters
H-atom parameters constrained
Δρ_max_ = 0.33 e Å^−3^
Δρ_min_ = −0.35 e Å^−3^

### 

Data collection: *CAD-4 Software* (Enraf–Nonius, 1989 ▶); cell refinement: *CAD-4 Software*; data reduction: *XCAD4* (Harms & Wocadlo, 1995 ▶); program(s) used to solve structure: *SHELXS97* (Sheldrick, 2008 ▶); program(s) used to refine structure: *SHELXL97* (Sheldrick, 2008 ▶); molecular graphics: *SHELXTL* (Sheldrick, 2008 ▶) and *PLATON* (Spek, 2003 ▶); software used to prepare material for publication: *SHELXTL*.

## Supplementary Material

Crystal structure: contains datablocks global, I. DOI: 10.1107/S1600536808028158/hk2521sup1.cif

Structure factors: contains datablocks I. DOI: 10.1107/S1600536808028158/hk2521Isup2.hkl

Additional supplementary materials:  crystallographic information; 3D view; checkCIF report

## Figures and Tables

**Table 1:** Hydrogen-bond geometry (Å, °)

*D*—H⋯*A*	*D*—H	H⋯*A*	*D*⋯*A*	*D*—H⋯*A*
C7—H7*B*⋯F2	0.97	2.34	3.002 (6)	124
C9—H9*A*⋯O1^i^	0.93	2.41	3.338 (5)	173

Symmetry code: (i) 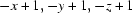.

**Fig. 1.**
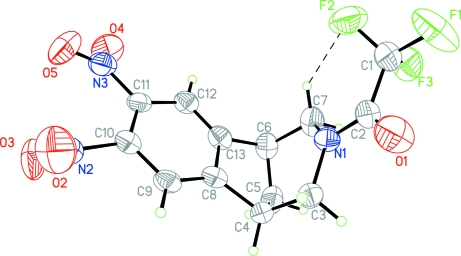
The molecular structure of the title molecule, with the atom-numbering scheme. Displacement ellipsoids are drawn at the 30% probability level. Hydrogen bond is shown as dashed line.

**Fig. 2.**
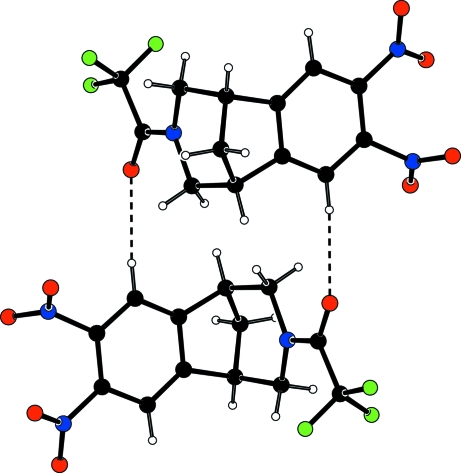
A partial packing diagram of the title compound. Hydrogen bonds are shown as dashed lines.

## supplementary crystallographic information

### Comment

Some derivatives of andrographolide are important chemical materials. We report herein the crystal structure of the title compound.

In the molecule of the title compound, (Fig. 1), the bond lengths (Allen *et al.*,  1987) and angles are generally within normal ranges. Ring A (C8–C13) is, of course, planar. Ring B (N1/C3–C7) is not planar, having total puckering amplitude, Q_T_, of 0.626 (3) and chair conformation [φ = -175.64 (3)° and θ = 36.12 (3)°] (Cremer & Pople, 1975), while ring C (C4–C6/C8/C13) adopts envelope conformation, with C5 atom displaced by -0.707 (3) Å from the plane of the other ring atoms. The intramolecular C—H···F hydrogen bond (Table 1) results in the formation of a nonplanar six-membered ring D (F2/C1/C2/N1/C7/H7B), having total puckering amplitude, Q_T_, of 0.651 (3) and twisted conformation [φ = 34.96 (3)° and θ = 76.80 (2)°] (Cremer & Pople, 1975)

In the crystal structure, intermolecular C—H···O hydrogen bonds (Table 1) link the molecules into centrosymmetric dimers (Fig. 2), in which they may be effective in the stabilization of the structure.

### Experimental

For the preparation of the title compound, 1-(4,5-diamino-10-aza-tricyclo- [6.3.1.0]dodeca-2,4,6-trien-10-yl)-2,2,2-trifluoro-ethanone (3.0 g) was hydrogenated in methanol (30 ml, 95%) under hydrogen (45 psi) over Pd-carbon catalysts (300 mg of 20wt%/C,10%wt). After 2.5 h, the reaction was filtered through a celite pad and rinsed with methanol (30 ml, 95%). The solution was concentrated to a light brown oil that crystallized. An X-ray grade crystal of the title compound (500 mg) was grown from ethyl acetate (10 ml) at room temperature.

### Refinement

H atoms were positioned geometrically, with C—H = 0.93, 0.98 and 0.97 Å for aromatic, methine and methylene H, respectively, and constrained to ride on their parent atoms with U_iso_(H) = 1.2U_eq_(C).

### Crystal data

**Table:**

C_13_H_10_F_3_N_3_O_5_	*F*_000_ = 704
*M**_r_* = 345.24	*D*_x_ = 1.656 Mg m^−^^3^
Monoclinic, *P*2_1_/*c*	Mo *K*α radiation λ = 0.71073 Å
Hall symbol: -P 2ybc	Cell parameters from 25 reflections
*a* = 9.6400 (19) Å	θ = 10–13º
*b* = 7.7430 (15) Å	µ = 0.15 mm^−^^1^
*c* = 18.687 (4) Å	*T* = 294 (2) K
β = 96.98 (3)º	Red, colorless
*V* = 1384.5 (5) Å^3^	0.30 × 0.20 × 0.10 mm
*Z* = 4	

### Data collection

**Table:**

Enraf–Nonius CAD-4 diffractometer	*R*_int_ = 0.052
Radiation source: fine-focus sealed tube	θ_max_ = 25.3º
Monochromator: graphite	θ_min_ = 2.1º
*T* = 294(2) K	*h* = −11→11
ω/2θ scans	*k* = 0→9
Absorption correction: ψ scan(North *et al.*, 1968)	*l* = 0→22
*T*_min_ = 0.955, *T*_max_ = 0.985	3 standard reflections
2673 measured reflections	every 120 min
2513 independent reflections	intensity decay: none
1592 reflections with *I* > 2σ(*I*)	

### Refinement

**Table:**

Refinement on *F*^2^	Secondary atom site location: difference Fourier map
Least-squares matrix: full	Hydrogen site location: inferred from neighbouring sites
*R*[*F*^2^ > 2σ(*F*^2^)] = 0.061	H-atom parameters constrained
*wR*(*F*^2^) = 0.183	*w* = 1/[σ^2^(*F*_o_^2^) + (0.05*P*)^2^ + 4*P*] where *P* = (*F*_o_^2^ + 2*F*_c_^2^)/3
*S* = 1.02	(Δ/σ)_max_ < 0.001
2513 reflections	Δρ_max_ = 0.33 e Å^−^^3^
217 parameters	Δρ_min_ = −0.35 e Å^−^^3^
Primary atom site location: structure-invariant direct methods	Extinction correction: none

### Special details

**Table:**

Geometry. All e.s.d.'s (except the e.s.d. in the dihedral angle between two l.s. planes) are estimated using the full covariance matrix. The cell e.s.d.'s are taken into account individually in the estimation of e.s.d.'s in distances, angles and torsion angles; correlations between e.s.d.'s in cell parameters are only used when they are defined by crystal symmetry. An approximate (isotropic) treatment of cell e.s.d.'s is used for estimating e.s.d.'s involving l.s. planes.
Refinement. Refinement of *F*^2^ against ALL reflections. The weighted *R*-factor *wR* and goodness of fit *S* are based on *F*^2^, conventional *R*-factors *R* are based on *F*, with *F* set to zero for negative *F*^2^. The threshold expression of *F*^2^ > σ(*F*^2^) is used only for calculating *R*-factors(gt) *etc*. and is not relevant to the choice of reflections for refinement. *R*-factors based on *F*^2^ are statistically about twice as large as those based on *F*, and *R*- factors based on ALL data will be even larger.

### Fractional atomic coordinates and isotropic or equivalent isotropic displacement parameters (Å^2^)

**Table:**

	*x*	*y*	*z*	*U*_iso_*/*U*_eq_	
F1	0.3302 (4)	0.7803 (5)	0.23330 (19)	0.1036 (13)	
F2	0.1596 (3)	0.6268 (5)	0.25814 (17)	0.0907 (12)	
F3	0.3030 (3)	0.5239 (4)	0.19179 (13)	0.0702 (9)	
O1	0.4775 (4)	0.6527 (5)	0.34298 (19)	0.0681 (10)	
O2	0.1222 (4)	0.4522 (5)	0.6478 (2)	0.0812 (12)	
O3	0.0247 (4)	0.2030 (5)	0.65267 (18)	0.0733 (11)	
O4	−0.2298 (3)	0.1740 (6)	0.4660 (2)	0.0772 (12)	
O5	−0.1736 (4)	0.3629 (5)	0.5498 (2)	0.0749 (11)	
N1	0.3630 (3)	0.3999 (5)	0.34108 (16)	0.0398 (8)	
N2	0.0824 (4)	0.3155 (6)	0.6213 (2)	0.0512 (10)	
N3	−0.1427 (4)	0.2615 (5)	0.5031 (2)	0.0532 (10)	
C1	0.2916 (5)	0.6217 (7)	0.2505 (2)	0.0557 (12)	
C2	0.3865 (4)	0.5564 (6)	0.3165 (2)	0.0446 (10)	
C3	0.4621 (4)	0.3471 (7)	0.4042 (2)	0.0486 (11)	
H3A	0.4714	0.4406	0.4390	0.058*	
H3B	0.5532	0.3272	0.3887	0.058*	
C4	0.4159 (4)	0.1853 (6)	0.4403 (2)	0.0442 (10)	
H4A	0.4872	0.1443	0.4784	0.053*	
C5	0.3737 (5)	0.0456 (7)	0.3846 (3)	0.0569 (12)	
H5A	0.3600	−0.0649	0.4072	0.068*	
H5B	0.4419	0.0331	0.3509	0.068*	
C6	0.2364 (4)	0.1203 (6)	0.3485 (2)	0.0476 (11)	
H6A	0.1799	0.0323	0.3208	0.057*	
C7	0.2679 (5)	0.2734 (7)	0.3008 (2)	0.0524 (12)	
H7A	0.3105	0.2310	0.2598	0.063*	
H7B	0.1811	0.3304	0.2827	0.063*	
C8	0.2766 (4)	0.2163 (5)	0.4683 (2)	0.0393 (9)	
C9	0.2468 (4)	0.2693 (5)	0.5352 (2)	0.0398 (10)	
H9A	0.3181	0.2968	0.5715	0.048*	
C10	0.1095 (4)	0.2805 (5)	0.5470 (2)	0.0391 (9)	
C11	0.0006 (4)	0.2439 (5)	0.4922 (2)	0.0381 (9)	
C12	0.0337 (4)	0.1944 (6)	0.4239 (2)	0.0417 (10)	
H12A	−0.0365	0.1737	0.3861	0.050*	
C13	0.1695 (4)	0.1778 (5)	0.4143 (2)	0.0418 (10)	

### Atomic displacement parameters (Å^2^)

**Table:**

	*U*^11^	*U*^22^	*U*^33^	*U*^12^	*U*^13^	*U*^23^
F1	0.150 (3)	0.070 (2)	0.083 (2)	0.000 (2)	−0.017 (2)	0.0327 (19)
F2	0.0614 (19)	0.134 (3)	0.076 (2)	0.042 (2)	0.0060 (15)	0.020 (2)
F3	0.0736 (19)	0.102 (2)	0.0356 (14)	0.0003 (17)	0.0089 (12)	−0.0006 (15)
O1	0.072 (2)	0.053 (2)	0.074 (2)	−0.0176 (19)	−0.0145 (18)	0.0037 (18)
O2	0.100 (3)	0.075 (3)	0.068 (2)	−0.011 (2)	0.005 (2)	−0.030 (2)
O3	0.095 (3)	0.076 (3)	0.056 (2)	−0.005 (2)	0.0403 (19)	0.009 (2)
O4	0.0338 (17)	0.108 (3)	0.087 (3)	−0.016 (2)	−0.0066 (17)	−0.001 (2)
O5	0.054 (2)	0.078 (3)	0.098 (3)	0.0127 (19)	0.029 (2)	−0.007 (2)
N1	0.0348 (17)	0.053 (2)	0.0291 (16)	−0.0060 (16)	−0.0073 (13)	−0.0019 (15)
N2	0.050 (2)	0.056 (3)	0.045 (2)	0.007 (2)	−0.0007 (17)	−0.003 (2)
N3	0.043 (2)	0.055 (3)	0.060 (2)	0.0015 (19)	0.0023 (19)	0.018 (2)
C1	0.054 (3)	0.067 (3)	0.046 (3)	0.004 (3)	0.008 (2)	0.014 (2)
C2	0.045 (2)	0.051 (3)	0.039 (2)	0.002 (2)	0.0101 (18)	0.007 (2)
C3	0.033 (2)	0.069 (3)	0.042 (2)	−0.007 (2)	−0.0008 (17)	0.009 (2)
C4	0.033 (2)	0.058 (3)	0.042 (2)	0.007 (2)	0.0055 (17)	0.010 (2)
C5	0.061 (3)	0.052 (3)	0.063 (3)	0.004 (2)	0.027 (2)	0.003 (2)
C6	0.049 (2)	0.053 (3)	0.041 (2)	−0.013 (2)	0.0063 (19)	−0.007 (2)
C7	0.053 (3)	0.071 (3)	0.033 (2)	−0.016 (2)	0.0025 (19)	−0.009 (2)
C8	0.041 (2)	0.038 (2)	0.037 (2)	0.0014 (18)	−0.0044 (17)	0.0045 (18)
C9	0.036 (2)	0.045 (2)	0.034 (2)	−0.0047 (19)	−0.0095 (16)	0.0003 (18)
C10	0.041 (2)	0.039 (2)	0.035 (2)	−0.0009 (18)	−0.0026 (17)	0.0051 (18)
C11	0.0318 (19)	0.038 (2)	0.042 (2)	0.0017 (17)	−0.0023 (16)	0.0104 (18)
C12	0.038 (2)	0.045 (3)	0.039 (2)	−0.0061 (19)	−0.0050 (17)	0.0008 (19)
C13	0.047 (2)	0.039 (2)	0.038 (2)	−0.0066 (19)	−0.0014 (18)	0.0015 (19)

### Geometric parameters (Å, °)

**Table:**

F1—C1	1.334 (6)	C4—C5	1.521 (6)
F2—C1	1.298 (5)	C4—H4A	0.9800
F3—C1	1.348 (6)	C5—C6	1.525 (6)
O1—C2	1.210 (5)	C5—H5A	0.9700
O2—N2	1.211 (5)	C5—H5B	0.9700
O3—N2	1.221 (5)	C6—C13	1.523 (6)
O4—N3	1.227 (5)	C6—C7	1.535 (6)
O5—N3	1.236 (5)	C6—H6A	0.9800
N1—C2	1.325 (6)	C7—H7A	0.9700
N1—C3	1.482 (5)	C7—H7B	0.9700
N1—C7	1.483 (5)	C8—C9	1.379 (6)
N2—C10	1.470 (5)	C8—C13	1.385 (5)
N3—C11	1.427 (5)	C9—C10	1.370 (5)
C1—C2	1.530 (6)	C9—H9A	0.9300
C3—C4	1.516 (6)	C10—C11	1.403 (5)
C3—H3A	0.9700	C11—C12	1.407 (6)
C3—H3B	0.9700	C12—C13	1.349 (6)
C4—C8	1.519 (5)	C12—H12A	0.9300
			
C2—N1—C3	114.0 (3)	C4—C5—H5B	111.7
C2—N1—C7	123.4 (3)	C6—C5—H5B	111.7
C3—N1—C7	121.4 (4)	H5A—C5—H5B	109.5
O2—N2—O3	124.6 (4)	C13—C6—C5	100.5 (3)
O2—N2—C10	117.6 (4)	C13—C6—C7	112.1 (4)
O3—N2—C10	117.8 (4)	C5—C6—C7	109.1 (4)
O4—N3—O5	122.9 (4)	C13—C6—H6A	111.5
O4—N3—C11	118.3 (4)	C5—C6—H6A	111.5
O5—N3—C11	118.8 (4)	C7—C6—H6A	111.5
F2—C1—F1	107.7 (4)	N1—C7—C6	111.6 (3)
F2—C1—F3	106.5 (4)	N1—C7—H7A	109.3
F1—C1—F3	105.6 (4)	C6—C7—H7A	109.3
F2—C1—C2	114.8 (4)	N1—C7—H7B	109.3
F1—C1—C2	110.0 (4)	C6—C7—H7B	109.3
F3—C1—C2	111.8 (4)	H7A—C7—H7B	108.0
O1—C2—N1	124.6 (4)	C9—C8—C13	120.4 (4)
O1—C2—C1	117.3 (4)	C9—C8—C4	130.6 (4)
N1—C2—C1	118.1 (4)	C13—C8—C4	109.1 (4)
N1—C3—C4	112.7 (3)	C10—C9—C8	118.4 (3)
N1—C3—H3A	109.0	C10—C9—H9A	120.8
C4—C3—H3A	109.0	C8—C9—H9A	120.8
N1—C3—H3B	109.0	C9—C10—C11	121.5 (4)
C4—C3—H3B	109.0	C9—C10—N2	116.7 (3)
H3A—C3—H3B	107.8	C11—C10—N2	121.6 (4)
C3—C4—C8	110.0 (4)	C10—C11—C12	119.0 (4)
C3—C4—C5	110.6 (3)	C10—C11—N3	121.9 (4)
C8—C4—C5	100.0 (3)	C12—C11—N3	119.0 (4)
C3—C4—H4A	111.9	C13—C12—C11	118.4 (4)
C8—C4—H4A	111.9	C13—C12—H12A	120.8
C5—C4—H4A	111.9	C11—C12—H12A	120.8
C4—C5—C6	100.3 (4)	C12—C13—C8	122.2 (4)
C4—C5—H5A	111.7	C12—C13—C6	130.3 (4)
C6—C5—H5A	111.7	C8—C13—C6	107.5 (4)
			
C3—N1—C2—O1	−1.8 (6)	C4—C8—C9—C10	−177.4 (4)
C7—N1—C2—O1	−169.0 (4)	C8—C9—C10—C11	−1.7 (6)
C3—N1—C2—C1	178.8 (4)	C8—C9—C10—N2	172.3 (4)
C7—N1—C2—C1	11.6 (6)	O2—N2—C10—C9	63.6 (5)
F2—C1—C2—O1	−121.0 (5)	O3—N2—C10—C9	−114.5 (5)
F1—C1—C2—O1	0.6 (6)	O2—N2—C10—C11	−122.3 (5)
F3—C1—C2—O1	117.5 (5)	O3—N2—C10—C11	59.5 (6)
F2—C1—C2—N1	58.5 (6)	C9—C10—C11—C12	0.1 (6)
F1—C1—C2—N1	−179.9 (4)	N2—C10—C11—C12	−173.6 (4)
F3—C1—C2—N1	−63.0 (5)	C9—C10—C11—N3	−177.6 (4)
C2—N1—C3—C4	168.3 (4)	N2—C10—C11—N3	8.7 (6)
C7—N1—C3—C4	−24.2 (5)	O4—N3—C11—C10	−154.4 (4)
N1—C3—C4—C8	−61.6 (5)	O5—N3—C11—C10	25.1 (6)
N1—C3—C4—C5	48.0 (5)	O4—N3—C11—C12	28.0 (6)
C3—C4—C5—C6	−71.8 (4)	O5—N3—C11—C12	−152.6 (4)
C8—C4—C5—C6	44.2 (4)	C10—C11—C12—C13	2.4 (6)
C4—C5—C6—C13	−44.6 (4)	N3—C11—C12—C13	−179.8 (4)
C4—C5—C6—C7	73.4 (4)	C11—C12—C13—C8	−3.4 (6)
C2—N1—C7—C6	−167.6 (4)	C11—C12—C13—C6	177.3 (4)
C3—N1—C7—C6	26.0 (5)	C9—C8—C13—C12	1.8 (7)
C13—C6—C7—N1	58.8 (5)	C4—C8—C13—C12	−179.6 (4)
C5—C6—C7—N1	−51.7 (5)	C9—C8—C13—C6	−178.8 (4)
C3—C4—C8—C9	−93.2 (5)	C4—C8—C13—C6	−0.2 (5)
C5—C4—C8—C9	150.4 (5)	C5—C6—C13—C12	−152.3 (5)
C3—C4—C8—C13	88.4 (4)	C7—C6—C13—C12	91.9 (5)
C5—C4—C8—C13	−28.0 (4)	C5—C6—C13—C8	28.3 (5)
C13—C8—C9—C10	0.8 (6)	C7—C6—C13—C8	−87.5 (4)

### Hydrogen-bond geometry (Å, °)

**Table:**

*D*—H···*A*	*D*—H	H···*A*	*D*···*A*	*D*—H···*A*
C7—H7B···F2	0.97	2.34	3.002 (6)	124
C9—H9A···O1^i^	0.93	2.41	3.338 (5)	173

Symmetry codes: (i) −*x*+1, −*y*+1, −*z*+1.
